# The impact of cancer information overload on negative emotions in newly diagnosed lung cancer patients: the mediating role of fear of progression and the moderating role of social support

**DOI:** 10.3389/fpsyt.2025.1676219

**Published:** 2025-10-29

**Authors:** Fan Xu, Jiquan Zhang, Qiao Li, Jing Wang, Ping Zhang, Weimin Dai, Shaoju Xie, Xiaoli Zhong

**Affiliations:** ^1^ Oncology Department, Deyang People’s Hospital, Deyang, Sichuan, China; ^2^ Nursing Department, Deyang People’s Hospital, Deyang, Sichuan, China

**Keywords:** lung cancer, cancer information overload, fear of progression, negative emotion, mediating effect

## Abstract

**Background:**

The high incidence of negative emotions among lung cancer patients has become a significant challenge to global public health. Newly diagnosed patients may experience cancer information overload (CIO) when exposed to a large amount of uncertain cancer-related information within a short time. However, research on the relationship between CIO and negative emotions, and the roles of Fear of Progression (FoP) and social support, is still lacking.

**Objective:**

To explore the mediating effect of FoP between CIO and negative emotion in newly diagnosed lung cancer patients, and the moderating role of social support among CIO, FoP and negative emotion.

**Method:**

This study adopted a cross-sectional survey design. From October 2024 to February 2025, newly diagnosed lung cancer patients were recruited as research subjects from the oncology departments of three tertiary hospitals in Deyang City, China. Through the General Information Questionnaire, Cancer Information Overload Scale (CIOS), Fear of Progression Questionnaire-Short Form (FoP-Q-SF), Hospital Anxiety and Depression Scale (HADS) and the Social Support Rating Scale (SSRS) were used to investigate patients’ CIO, FoP, negative emotions and social support, and a total of 358 valid questionnaires were retrieved. All data were processed using SPSS 26.0, and the mediating effect and moderating effect were tested using the Process plugin.

**Results:**

The incidence of anxiety was 56.1% (201/358), and the incidence of depression was 53.1% (190/358). CIO had a positive predictive effect on FoP (β=0.338, *P* < 0.001) and negative emotion (β=0.375, *P* < 0.001). FoP has a positive predictive effect on negative emotion (β=0.342, *P* < 0.001), and FoP plays a mediating role between CIO and negative emotion (β= 0.115, 95%CI=[0.072, 0.166]). Social support plays a negative moderating role between FoP and negative emotion, with a moderating index of -0.124 and 95%CI=[-0.214,-0.033].

**Conclusion:**

Patients with newly diagnosed lung cancer bear a heavy burden of anxiety and depression, which urgently needs attention. CIO plays a mediating role between FoP and negative emotion. Social support weakens the positive predictive effect of FoP on negative emotion. Our research results provide new insights and methods for supporting the improvement of negative emotions in lung cancer patients.

## 1 Introduction

Global cancer statistics show that in 2022, there were 2.48 million new cases of lung cancer, accounting for 12.4% of all new cancer cases, making it the most common cancer worldwide (1). Owing to the insidious onset of lung cancer, the lack of effective screening methods, and the absence of specific early - stage symptoms, patients often present at clinics in the middle or late stages of the disease. Additionally, the age - standardized 5 - year relative survival rate for lung cancer is typically low, ranging from 10% to 20% in most regions (2). Under the dual pressures of high incidence and low survival rates, negative emotions among lung cancer patients have become increasingly prominent, posing a major challenge to global public health. In the assessment and intervention of mental health, anxiety and depression are frequently utilized as core indicators for negative emotions in research and measurement (3). Studies have shown that the incidence of anxiety among lung cancer patients ranges from 30% to 43.5%, while the incidence of depression ranges from 32.4% to 57.1% (4–7). Anxiety and depression not only affect patients’ quality of life (8),it may also lead to obstacles in cancer treatment decisions, poor treatment compliance, and prolonged recovery times (9, 10), even leads to an increased risk of cancer-specific mortality and all-cause mortality in patients (11). Therefore, in-depth research into the causes and impact mechanisms of negative emotions, especially anxiety and depression, in lung cancer patients is of great practical significance and urgency.

Cancer Information Overload (CIO) is a construct defined as “feeling overwhelmed by the amount of cancer-related material in the information environment” (12). In the early stages of lung cancer diagnosis, patients are inundated with a large amount of information in a short period of time, including complex medical terminology that is difficult to understand. Patients are overwhelmed by disease information, treatment options, prognosis details, and other data, making it challenging to effectively filter and utilize this information, often leading to CIO. Additionally, research has shown that 70.6% of brain tumor patients use online platforms to learn about the latest medical and scientific information related to cancer (13),80.2% of cancer patients use social media to connect with others to learn about cancer (14). Although these channels provide patients with abundant information resources and communication platforms, enabling them to obtain disease knowledge, treatment experiences, and emotional support, the vastness and complexity of information may also lead to CIO.

The Limited Capacity Model of Motivated Mediated Message Processing (LC4MP) posits that individuals possess finite information - processing capacity, which is influenced by diverse motivations, including self - defense and information - processing motives (15, 16). When the pace of information acquisition surpasses an individual’s information - processing capacity and absorption threshold, these motives may be activated or disrupted. Patients, driven by self - defensive motives, may selectively ignore certain information, or due to disrupted information - processing motives, may give up trying to understand it. This doesn’t only notably reduce information - processing efficiency, but also exerts profound negative impacts on an individual’s emotional state, social relationships, as well as physical and mental health (15, 16). At the same time, when faced with excessive, complex, or contradictory information about diseases, individuals are prone to cognitive and mental confusion, which can lead to psychological and emotional disorders (17). Therefore, CIO may trigger or exacerbate negative emotions in patients.

Research shows that CIO may cause patients to experience negative psychological reactions such as breakdowns and panic, exacerbating Fear of Progression (FoP) (18). Moreover, Elevated levels of CIO can evoke a spectrum of physical and emotional responses in patients when they face treatment decisions (19), with FoP being the most prevalent (20). Two distinct surveys of head and neck cancer and esophageal cancer patients demonstrated a positive correlation between CIO and FoP (21, 22), as information overload leaves patients feeling bewildered and helpless when confronted with excessive information, thereby intensifying their anxiety, which predominantly manifests as intense anxiety regarding disease recurrence, metastasis, or progression. Such excessive worry can impose persistent psychological stress on patients, potentially triggering anxiety symptoms. If left unaddressed, it may further evolve into depression. Research indicates that FOP in cancer patients is a significant factor influencing anxiety and depression, exhibiting a positive correlation with these conditions (23). Among nasopharyngeal carcinoma patients, those in the high FoP group had a 16.34-fold increased risk of developing moderate to severe anxiety and a 6.9-fold increased risk of developing moderate to severe depression and anxiety simultaneously (24). Overall, CIO increased patients’ FoP, and FoP had a significant impact on patients’ negative emotions. Therefore, we speculate that fear of cancer progression may act as a mediating variable between CIO and negative emotions.

In addition, this study introduced social support as a moderating variable. Social support refers to the emotional and material support that individuals obtain from their social relationships. It includes the sum of emotional, informational, or practical help that individuals perceive from significant others (25). The buffering effect model of social support suggests that social support mitigates the negative impact of stress on physical and mental health by regulating the cognitive evaluation and coping process of stressful events (26). Based on this model, we hypothesize that social support can alleviate the psychological stress caused by CIO and FoP in patients with newly diagnosed lung cancer and improve their negative emotions. A survey of medical students showed that social support alleviated the impact of stressors on negative emotions (27). Moreover, previously research indicates that social support, as an external coping resource, not only assists patients in more effectively addressing disease challenges by providing emotional comfort, practical assistance, and informational resources but also promotes positive cognitive restructuring and strengthens stress-coping mechanisms, thereby alleviating FoP (28, 29).Therefore, social support may play a moderating role between CIO and FoP and Negative emotions, that is, higher social support can buffer the impact of CIO and FoP on Negative emotions.

**Figure 1:**
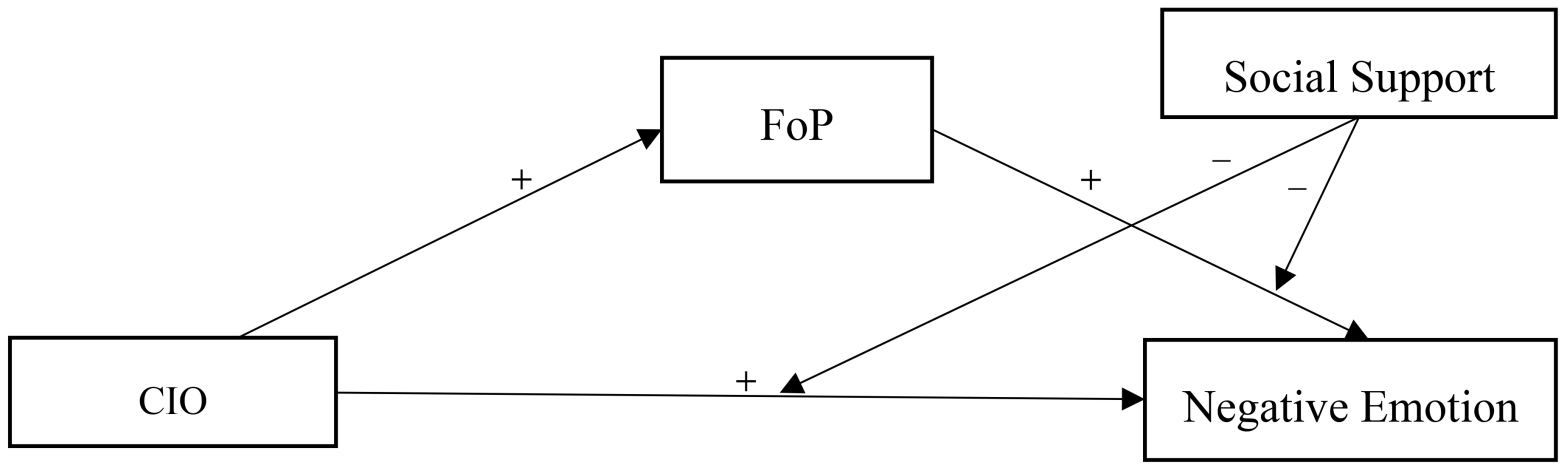
The proposed mediated moderation model. CIO, Cancer Information Overload; FoP, Fear of Progression.

Currently, most research has primarily focused on examining the pairwise relationships between CIO, FoP, social support, and Negative Emotion (30–32), without yet establishing a systematic analysis of the interactions among these factors. Based on the LC4MP and the buffering effect model of social support, this study uses anxiety and depression as negative emotion outcomes to explore a complex moderated mediation model underlying the association between CIO and negative emotions in patients newly diagnosed with lung cancer. The objectives of our study were to (a) examine whether CIO significantly and positively predicts negative emotion; (b) examine whether FoP mediates the relationship between CIO and negative emotion; (c) examine whether social support moderates the relationship between CIO, FoP, and negative emotion. The hypothetical model of this study is shown in **Figure 1**.

## 2 Method

### 2.1 Participants

This study employed a cross-sectional survey design. From October 2024 to February 2025, newly diagnosed lung cancer patients were recruited as study subjects from the oncology departments of three tertiary hospitals in Deyang City, China.

Inclusion criteria: (1) Age ≥ 18 years; (2) Pathologically diagnosed with lung cancer, with less than 6 months since diagnosis; (3) Possessing basic language comprehension and expression abilities; (4) Voluntarily participate and sign an informed consent form. Exclusion criteria: (1) Tumor metastasis or concomitant malignant tumors; (2) Presence of mental or cognitive impairments; (3) Lactation or pregnancy; (4) Patients receiving end-of-life care. This study has been approved by the Ethics Committee of Deyang People’s Hospital (Ethics Approval Number: 202404077K01).

**Figure 2:**
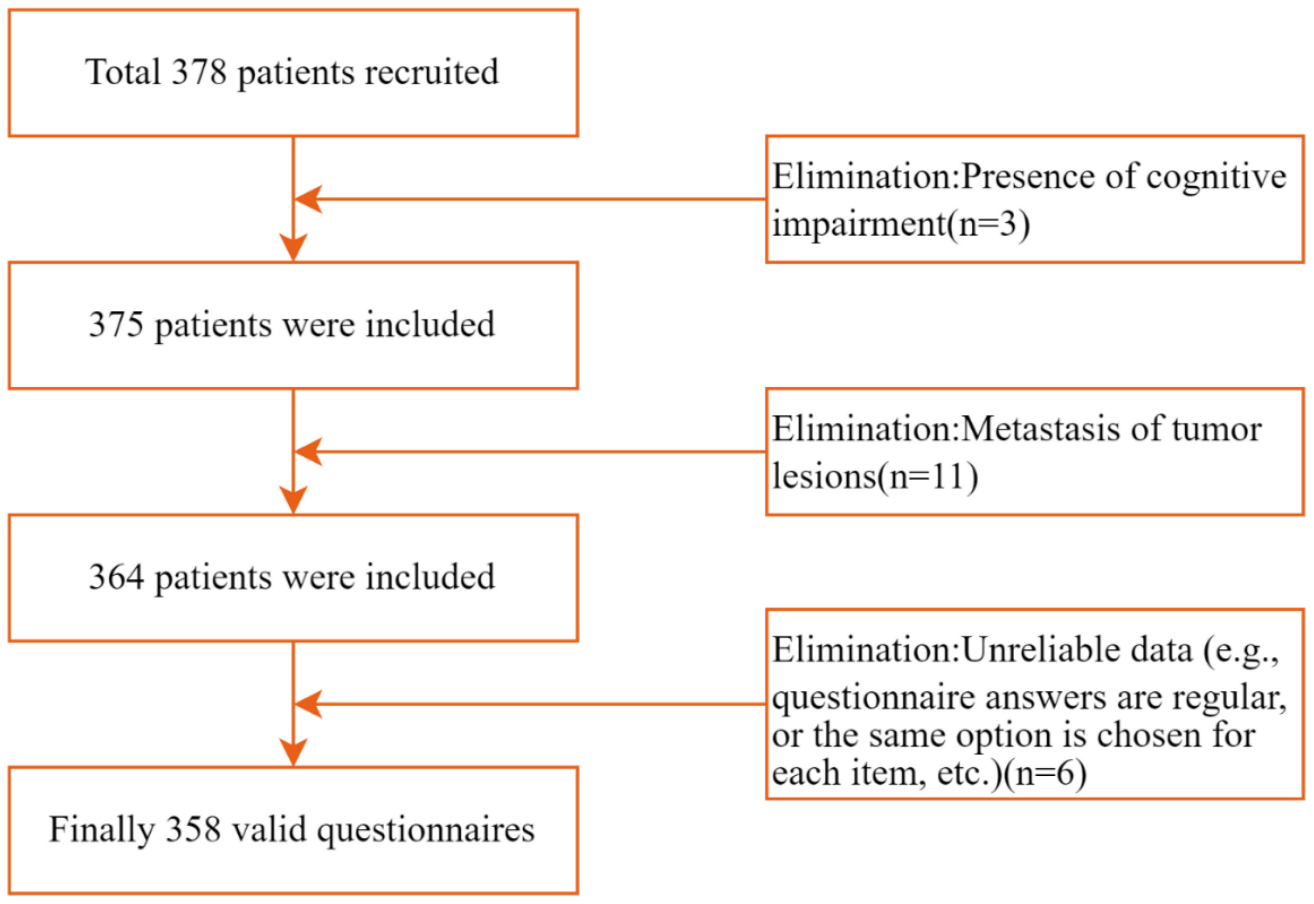
The process of participant selection.

Based on the linear regression model, the required sample size was calculated using G*Power software (Version 3.1) (33), set Power to 0.95 and Alpha to 0.05 according to Cohen’s standard (34)., Assuming an effect size f^2^ of 0.15 (moderate effect size) and 17 independent variables involved in this study [(including CIO, FoP, Social Support, and 14 variables covered in demographic and disease-related data)], G*Power calculated the required sample size to be 208. Considering a 10% attrition rate, the estimated minimum sample size required is 229 cases. A total of 378 lung cancer patients were recruited to complete the questionnaire. Three patients were excluded due to cognitive impairments, and 11 patients were excluded due to tumor metastasis. After the questionnaires were returned, six were excluded due to unreliable data (e.g., consistent patterns in responses or identical selections for each item). Ultimately, 358 valid questionnaires were recovered, yielding an effective recovery rate of 94.9%. **Figure 2** illustrates the participant selection process.

### 2.2 Measures

#### 2.2.1 General information

This includes demographic data and disease-related data. Demographic data includes age, marital status, employment status, Level of education, Average monthly family income, and Place of residence. Disease-related data includes Smoking history, Surgery and clinical stage, etc.

#### 2.2.2 Cancer information overload scale

This scale was developed by Jensen et al. in 2014 (12), and translated into Chinese by Ding Yao for use in assessing patients’ CIO status (35).This is a unidimensional scale with 8 items, for instance: ‘I haven’t had sufficient time to manage my cancer entirely according to the recommended advice’; ‘After a while, all the information about cancer seems to become indistinct’; ‘I feel I’ve received an excessive amount of information about cancer, surpassing what I wish to know’. The scale uses a 4-point Likert scale, ranging from “strongly disagree” to “strongly agree,” scored from 1 to 4 points, with total scores range of 8 to 32 points. Higher scores indicate higher levels of CIO. In this study, the Cronbach’s alpha coefficient was 0.806.

#### 2.2.3 Fear of Progression Questionnaire-Short Form

This questionnaire was developed by German scholars Mehnert et al. in 2006 based on the FoR-Q (36), with a Cronbach’s alpha coefficient of 0.87. Wu Qiyun et al. translated and revised it for use in China (37), The revised Chinese version comprises two dimensions: fear of physical health and fear of social and family issues, with 12 items,for instance: ‘The thought that my illness might progress makes me anxious’; ‘I feel nervous before doctor’s appointments or regular check-ups’; ‘The prospect of having to rely on strangers for my daily life causes me anxiety’. It uses a 5-point Likert scale, scored from 1 to 5 (never, rarely, sometimes, often, always), with total scores range of 12 to 60. Higher scores indicate greater fear of disease progression. A total score of ≥34 indicates reaching the threshold requiring clinical intervention. In this study, the Cronbach’s alpha coefficient was 0.812.

#### 2.2.4 Hospital anxiety and depression scale

Developed by Zigmond AS and Snaith RP in 1983 (38), and translated into Chinese by Ye Weifei et al. (39), this scale is used for screening anxiety and depression in patients in general hospitals. The scale consists of two dimensions: anxiety (HADS-A) and depression (HADS-D), with a total of 14 items, seven items for each dimension, for instance, ‘My heart is filled with worry’;’I suddenly felt a sense of panic’; ‘I seem to feel my mood gradually sinking’. Each item is scored on a 4-point scale from 0 to 3 based on the frequency of symptom occurrence over the past month. The total score ranges from 0 to 21 points, with scores of 0–7 indicating no cases, 8–10 indicating suspected cases, and 11–21 indicating confirmed cases (38).In this study, the Cronbach’s alpha coefficient was 0.864.

#### 2.2.5 Social support rating scale

Developed by Xiao Shuiyuan (40), this scale assesses an individual’s social support level through three dimensions: objective support, subjective support, and support utilization. It contains 10 items, for example: ‘How many close friends do you have who can offer support and assistance?’;’Do you actively confide in others when troubled?’;’What methods do you use to seek help when troubled?’. Items 1–4 and 8–10 are multiple - choice, scored 1–4. Item 5, with options A–E, is scored 1–4 from “none” to “full support”. For Items 6 and 7, “no sources” scores 0, while “the following sources” scores by the number of sources. The total score, the sum of all items, ranges from 12 to 66, with higher scores indicating greater social support. In this study, the Cronbach’s alpha coefficient was 0.819.

### 2.3 Data collection

Prior to the survey, researchers conducted standardized training for all participants, covering topics such as inclusion and exclusion criteria for research subjects, research objectives and content, and how to use the questionnaire. During the survey, investigators explained the study’s purpose and procedure to patients, obtained informed consent, and had patients fill out questionnaires anonymously and one - on - one. For patients with low literacy or advanced age who struggled to complete the questionnaire, investigators asked each question individually and recorded answers verbatim. They clarified questions for confused patients without suggesting answers. Completed questionnaires were collected and checked on the spot for omissions or errors. After data collection, two staff members verified and entered data into Excel, with a third checking for accuracy.

### 2.4 Data analyses

Statistical analysis was performed using SPSS 26.0. A common method bias test was conducted using exploratory factor analysis via Harman’s single - factor test. P-P and Q - Q plots were used to assess approximate normal distribution of the data. Descriptive statistics were presented as mean ± standard deviation. Pearson correlation analysis was used to examine the relationships between CIO, FoP, social support, and negative emotions. Using Model 4 (testing simple mediation) in Hayes’ PROCESS 4.1 macro program to analyze the mediating effect of FoP between CIO and negative emotion, Model 15 (testing moderated mediation) to analyze whether social support moderates the direct path between CIO and negative emotion, and the latter half of the mediating model path (41). Then, the simple slope method was used to analyze the moderating effect of social support on the relationship between CIO, FoP, and negative emotion. In the current study, there were statistically significant differences in the total scores of negative emotion among newly diagnosed lung cancer patients across employment status, surgery, radiotherapy, cancer stage, and the number of comorbid chronic diseases (F = 3.047, *P* = 0.029; t=-3.392, *P* = 0.001; t=2.749, *P* = 0.006; F = 3.183, *P* = 0.024; F = 4.635, *P* = 0.0210). Therefore, employment status, surgery, radiotherapy, cancer stage, and number of comorbidities were used as control variables. All tests used the bias-corrected percentile Bootstrap method, with 5,000 repeated samples, to calculate the 95% confidence interval. When the confidence interval did not include 0, the mediating effect, moderated mediating effect, and moderating effect were considered significant (42). The significance level is set at alpha = 0.05.

## 3 Results

### 3.1 Basic information

This study included 358 newly diagnosed lung cancer patients with an average age of 62.46 ± 11.78 years (range 35–85 years), with a majority being male (57.3%). The patients were predominantly married (67.0%), had a low to moderate level of education (34.4% with primary school education or below, 46.1% with secondary school education), and were rural residents (52.8%). The majority (89.9%) of patients had a monthly household income of ≤10,000 yuan. Employment status distribution was as follows: full-time 24.6%, part-time 20.4%, unemployed 34.1%, and retired 20.9%. The majority of patients had a history of smoking (81.0%). Cancer staging was concentrated in stages II-III (78.5%), and the prevalence of comorbidities was high (≥1 comorbidity in 73.5% of cases). In terms of treatment, surgery was the most widely used (71.5%), followed by radiotherapy (63.1%) and chemotherapy (52.0%).

### 3.2 Comparison of negative emotion scores among newly diagnosed lung cancer patients across different demographic characteristics

This study’s results indicate statistically significant variations in negative emotion scores among newly diagnosed lung cancer patients across factors like employment status, surgery history, radiotherapy, cancer stage, and number of comorbid chronic conditions (*P* < 0.05). Additionally, the prevalence of anxiety and depression was calculated, with anxiety affecting 56.1% (201/358) and depression affecting 53.1% (190/358) of participants. The details as shown in **Table 1**.

**Table 1:** Comparison of negative emotion scores across different demographic characteristics (n=358).

Characteristic	Item	Total Participants[n(%)]	HADS (M±SD)	*t/F* Value	*P* Value	LSD
Gender	Male	205(57.3)	21.55±7.71	-0.312	0.755	
	Female	153(42.7)	21.80±7.72			
Age (years)	<45	25(7.0)	23.16±5.23	1.008*	0.366	
	45–59	127(35.5)	21.01±7.39			
	≥60	206(57.5)	21.87±8.13			
Marital status	Unmarried	12(3.4)	20.17±6.98	0.266*	0.767	
	Married	247(70.0)	21.78±7.85			
	Divorced/Widowed	99(27.6)	21.54±7.47			
Level of education	Elementary school or below	123(34.4)	21.63±7.98	0.165*	0.848	
	Middle school	165(46.1)	21.48±7.69			
	College or above	70(19.5)	22.11±7.35			
Place of residence	Urban	169(47.2)	22.08±7.04	0.999	0.319	
	Rural	189(52.8)	21.28±8.25			
Average monthly family income (yuan)	<3,000	65(18.2)	22.97±7.21	2.498*	0.060	
	3,000–5,000	165(46.1)	22.24±8.00			
	5,001–10,000	92(25.7)	20.02±8.00			
	>10,000	36(10.1)	20.81±5.66			
Employment status	Full-time ^a^	88(24.6)	20.55±7.23	3.047*	0.029	a,b<c
	Part-time ^b^	73(20.4)	20.53±8.07			
	Unemployed ^c^	122(34.1)	23.30±7.77			
	Retired ^d^	75(20.9)	21.39±7.47			
Smoking history	Yes	290(81.0)	21.28±7.74	-1.902	0.058	
	No	68(19.0)	23.25±7.39			
Surgery	Yes	256(71.5)	20.80±7.88	-3.392	0.001	
	No	102(28.5)	23.81±6.81			
Chemotherapy	Yes	186(52.0)	22.08±7.61	1.083	0.279	
	No	172(48.0)	21.20±7.81			
Radiotherapy	Yes	226(63.1)	22.50±7.45	2.749	0.006	
	No	132(36.9)	20.2±7.95			
Cancer stage	Stage I ^~^ II	171(47.8)	20.57±7.40	-2.563	0.011	
	Stage III ^~^ IV	187(52.2)	22.65±7.87			
Number of chronic diseases	None ^a^	95(26.5)	20.66±7.35	4.635*	0.010	a,b<c
	1-2types ^b^	141(39.4)	20.85±7.41			
	≥3 types ^c^	122(34.1)	23.36±8.08			

*Indicates one-way analysis of variance.

a, b, c and d represent different category groups.

### 3.3 Common method biases test

Exploratory factor analysis was used in Harman’s single factor test to ensure the reliability and accuracy of the data. The results showed that there were 12 factors with characteristic root values greater than 1 without rotation, and the variance explained by the first factor was 16.26%, which was below the critical standard of 40%, indicating that there was no serious common method bias in the data of this study (43).

### 3.4 Scoring and correlation analysis

Newly diagnosed lung cancer patients showed a positive correlation between CIO and FoP (r = 0.355, *P* < 0.01) and negative emotion (r = 0.375, *P* < 0.01); FoP was positively correlated with negative emotion (r = 0.424, *P* < 0.01) and negatively correlated with social support (r = -0.161, *P* < 0.01); Negative emotion was negatively correlated with social support (r = -0.177, *P* < 0.01). (The details as shown in **Table 2**).

**Table 2:** Descriptive statistics and correlations for all variables.

Variables	M±SD	CIO	FoP	Negative emotion	Social support
CIO	20.46±4.87	1			
FoP	40.49±6.14	0.355**	1		
Negative emotion	21.66±7.71	0.375**	0.424**	1	
Social support	36.65±8.15	-0.016	-0.161**	-0.177**	1

^**^P < 0.01.

### 3.5 Mediation effect of FoP

The mediating effect of FoP between social support and negative emotion was tested using Model 4 in the PROCESS 4.1 macro program. After controlling for the effects of employment status, surgery, radiotherapy, cancer stage, and Number of chronic diseases, CIO in newly diagnosed lung cancer patients can positively predict negative emotion (β = 0.375, 95% CI [0.281, 0.470], *P* < 0.001), and positively predict FoP (β = 0.338, 95% CI [0.239, 0.437], *P* < 0.001); FoP positively predicted negative emotion in newly diagnosed lung cancer patients (β = 0.342, 95% CI [0.249, 0.435], *P* < 0.001), and the direct effect of CIO on negative emotion was also significant (β = 0.260, 95% CI [0.166, 0.353], *P* < 0.001). As shown in **Table 3**.

**Table 3:** Testing the mediating effect of FoP between CIO and negative emotion.

Variable	Model 1		Model 2		Model 3	
	Negative emotion		FoP		Negative emotion	
	*β*	*t*	*β*	*t*	*β*	*t*
Employment status	0.012	0.246	-0.046	-0.876	0.028	0.602
Surgery	-0.297	-2.495	-0.094	-0.748	-0.265	-2.381*
Radiotherapy	0.159	1.487	-0.004	-0.039	0.161	1.608
Cancer stage	0.112	1.719*	-0.041	-0.604	0.126	2.074*
Number of chronic diseases	0.146	2.281*	-0.021	-0.306	0.153	2.561*
CIO	0.375	7.844***	0.338	6.693***	0.260	5.472***
FoP	——	——	——	——	0.342	7.250***
R^2^	0.456		0.343		0.558	
Adjust R^2^	0.208		0.117		0.311	
*F*	15.343		7.778		22.592	

^*^p < 0.05, ^***^p < 0.001.

Further applying the bias-corrected Bootstrap method with 5,000 resampling iterations, the results indicate that CIO has a significant indirect effect on negative emotions in newly diagnosed lung cancer patients through FoP: β = 0.115, SE = 0.024, 95% CI = [0.072, 0.166]. The direct effect of CIO (0.260) and the mediating effect of FoP (0.115) account for 69.33% and 30.67% of the total effect, respectively. Therefore, the mediating role of FoP between CIO and negative emotion is validated. As shown in **Table 4**.

**Table 4:** Bootstrap based mediation effect test.

Effect type	*β*	BootSE	*t*	Boot LLCI	Boot ULCI	*P*	Effect Size (%)
Total effect	0.375	0.048	7.844	0.281	0.470	<0.001	100
Direct effect	0.260	0.047	5.472	0.166	0.353	<0.001	69.33
Indirect effect	0.115	0.024	——	0.072	0.166	——	30.67

### 3.6 Moderated mediation

**Figure 3:**
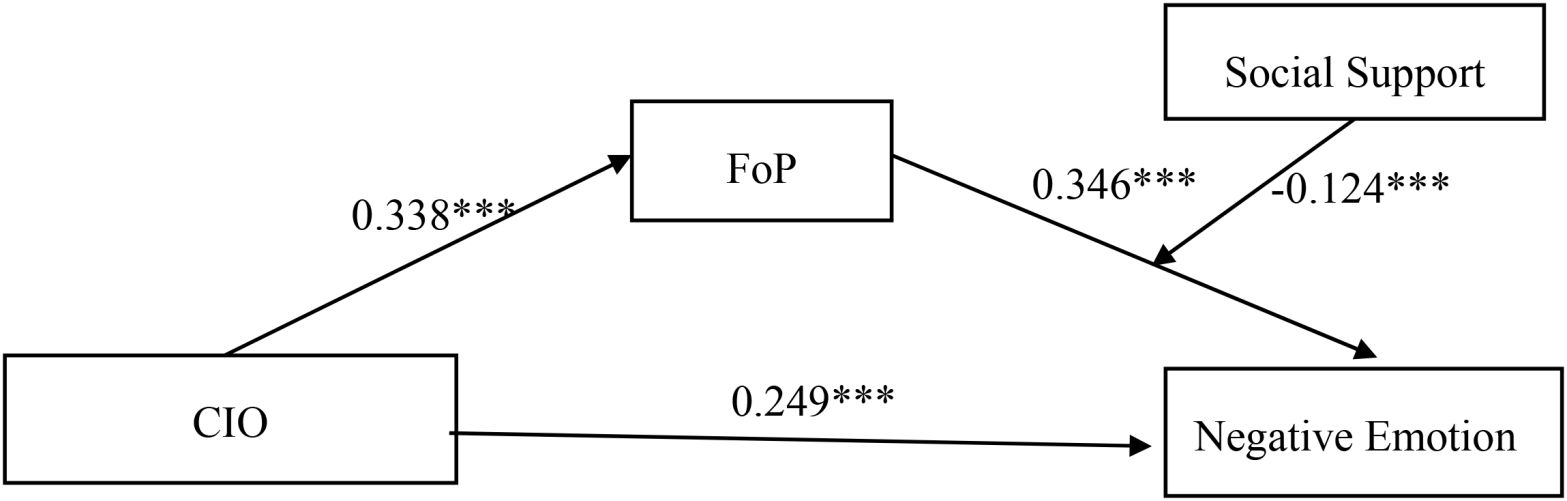
Mediated moderation model. ***P < 0.001. CIO, Cancer Information Overload; FoP, Fear of Progression.

The Model 15 test in the PROCESS 4.1 macro program was used to examine whether social support moderated the direct path between CIO and negative emotion, as well as the latter half of the mediating model. After controlling for the effects of employment status, surgery, radiotherapy, cancer stage, and number of chronic diseases, the interaction between CIO and social support on negative emotion in newly diagnosed lung cancer patients was not significant (β = 0.056, 95% CI [-0.039, 0.151], *P* = 0.248), while FoP and social support had a significant interaction effect on negative emotion in newly diagnosed lung cancer patients (β=-0.124, 95% CI [-0.214, -0.033], *P* = 0.008). As shown in **Table 5** and **Figure 3**.

**Table 5:** Testing moderated mediation effect of acceptance on social support and negative emotion.

Variable	Model 1		Model 2	
	FoP		Negative emotion	
	β	t	β	t
Employment status	-0.046	-0.876	0.020	0.434
Surgery	-0.094	-0.748	-0.285	-2.605*
Radiotherapy	-0.004	-0.039	0.173	1.740
Cancer stage	-0.041	-0.604	0.136	2.279*
Number of chronic diseases	-0.021	-0.306	0.164	2.800*
CIO	0.338	6.693***	0.249	5.295***
FoP	——	——	0.346	7.188***
Social support	——	——	-0.154	-3.447***
CIO×Social support			0.056	1.158
FoP×Social support	——	——	-0.124	-2.677**
*R* ^2^	0.343		0.589	
Adjust *R^2^*	0.117		0.347	
*F*	7.778		18.443	

^*^P < 0.05, ^**^P < 0.01, ^***^P < 0.001.

Using the bias-corrected Bootstrap method with 5,000 resampling iterations, the results showed that the 95% confidence interval for the interaction term between FoP and social support did not include 0 (95% CI = [-0.082, -0.010]), with a moderation index of -0.042 and SE = 0.018. When social support is low (below the mean by one standard deviation), FoP has a significant mediating effect between CIO and negative emotion in newly diagnosed lung cancer patients: β = 0.159, 95% CI = [0.087, 0.244]. Conversely, when social support is high (one standard deviation above the mean), FoP still has a significant mediating effect between CIO and negative emotion in newly diagnosed lung cancer patients: β = 0.075, 95% CI = [0.032, 0.120], the mediating effect value decreases, indicating that social support has a moderating effect on the indirect effects of CIO and negative emotion.

**Figure 4:**
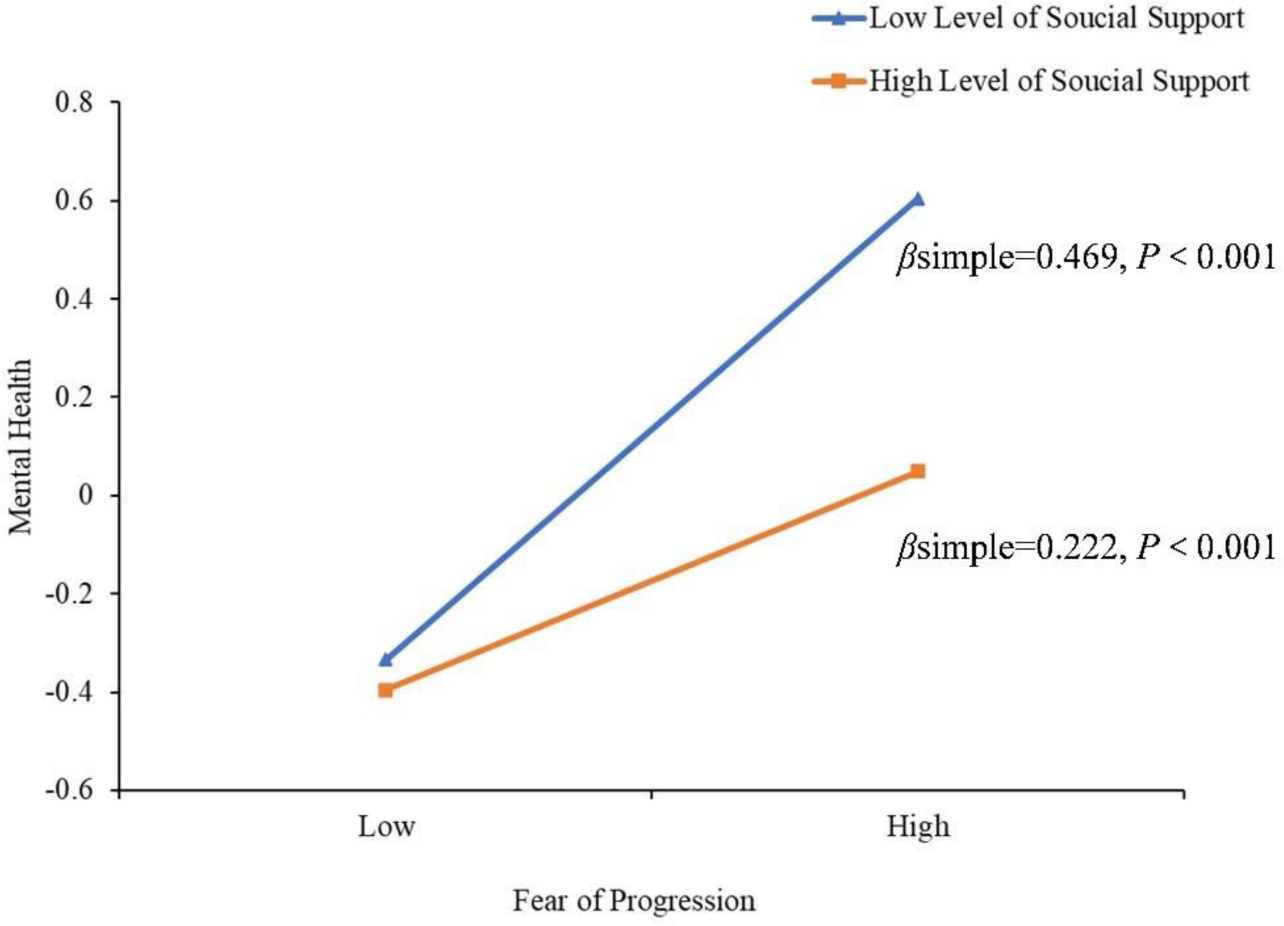
Model of test for simple slopes showing moderating influence of social support in the association between FoP and negative emotion.

To better understand the moderating effect of social support on FoP and negative emotion, a simple slope test was used for analysis. As shown in **Figure 4**, when social support was at a low level (M-1SD), the positive predictive effect of FoP on negative emotion was significant in newly diagnosed lung cancer patients (β simple = 0.469, *P* < 0.001). When social support is at a high level (M + 1SD), the positive predictive effect of FoP on negative emotion is also significant (β simple = 0.222, *P* < 0.001), but the predictive effect is relatively reduced. Therefore, as social support increases, the predictive effect of FoP on negative emotion shows a gradually decreasing trend, and social support plays a moderating role in the influence of FoP on negative emotion.

## 4 Discussion

With the rapid development of information technology, social media and diverse medical information channels have improved patients’ understanding of diseases, but have also increased the complexity of the information environment. For patients diagnosed with lung cancer for the first time, a large amount of uncertain and even contradictory cancer-related information in a short period of time can easily lead to CIO. This study is based on the limited capacity motivation media information processing model and the buffering effect model of social support. It comprehensively considers the mediating role of FoP and the moderating role of social support to deeply explore the impact mechanism of CIO on negative emotions such as anxiety and depression in patients with newly diagnosed lung cancer, providing new empirical evidence for understanding and intervening in patients’ psychological distress.

The results of this study indicate that among 358 newly diagnosed lung cancer patients, the incidence of anxiety reached 56.1%, and the incidence of depression was 53.1%, both higher than the rates of negative emotions reported in lung cancer patients from other countries (Mexico: anxiety 35%/depression 31%) (44), Poland(anxiety 37.2%/depression 41.7%) (45), and also higher than some domestic studies (anxiety 30%/depression 32.4%) (6). It may be due to the unique stigma associated with illness, communication barriers within families, and the absence of social support faced by Chinese patients, coupled with the systemic lack of psychological intervention within the healthcare system, which further exacerbates psychological distress. Additionally, this study focuses on the initial diagnosis stage of lung cancer, where the psychological impact of a cancer diagnosis and treatment uncertainty can trigger acute stress reactions, leading to a high incidence of negative emotions. Another domestic study on newly diagnosed lung cancer patients reported similar high levels of anxiety (57.57%) and depression (59.05%) (46), this highlights the significant psychological burden that Chinese lung cancer patients face at the time of diagnosis, underscoring the urgent need for heightened attention from both clinical and public health sectors.

The results of this study show that negative emotions differ significantly across demographic and disease characteristics such as employment status, surgery, radiotherapy, cancer stage, and number of chronic diseases. In terms of employment status, unemployed patients scored higher on negative emotions than full-time and part-time patients, consistent with prior research findings (47). A meta-analysis encompassing 237 cross-sectional and 87 longitudinal studies confirmed that unemployed individuals experience greater psychological distress than their employed counterparts (48). Consequently, the absence of stable social roles and economic income may render unemployed individuals more vulnerable when confronting illness, while simultaneously facing greater financial pressure regarding healthcare costs, thereby exacerbating negative emotions. In terms of treatment methods, patients who underwent surgery had lower negative emotion scores than those who did not undergo surgery, consistent with the findings of Astrup et al. (49). This may be because surgery is viewed as a direct means of removing the tumor, and patients tend to associate it with the “hope of cure,” which can provide them with the expectation of recovery and psychological comfort, thereby improving their mental state; However, some studies have also pointed out that surgery is a negative factor affecting patients’ anxiety and depression (50)., this discrepancy may be related to the patient’s baseline characteristics, the measurement tools employed, expectations regarding surgical outcomes, the course of postoperative recovery, and the quality of postoperative management. Moreover, patients undergoing radiotherapy exhibited significantly higher negative emotional scores than those not receiving radiotherapy, consistent with findings reported by Luo et al. (51). this may be attributable to the fact that radiotherapy is frequently associated with certain side effects, such as fatigue, nausea, and vomiting, which may lead to a decline in patients’ quality of life and negatively impact their mental health (52).

In addition, patients with stage III–IV lung cancer exhibited higher negative affect scores than those with stage I–II disease, indicating that disease severity directly exacerbates negative emotions, consistent with the results of similar studies (53), This may be related to the fact that patients with advanced lung cancer are prone to pessimistic cognition due to severe symptoms, complex treatment, and low survival expectations (11). In terms of the number of chronic diseases, patients with three or more chronic conditions had significantly higher negative emotion scores compared to those with none or one to two comorbidities. A previous retrospective cohort study also noted that the number of comorbid conditions was significantly associated with the risk of anxiety (adjusted odds ratio [95% confidence interval (CI)]: 1.17 [1.15–1.19]), and depression (1.24 [1.21–1.26]) (54), it may be that multiple chronic conditions exacerbate patients’ physiological burdens, leading to increased physical discomfort and medical needs, heightened concerns about health status and future uncertainties, and consequently triggering negative emotions (55, 56).

This study found that negative emotions in patients newly diagnosed with lung cancer were associated with higher levels of CIO, a finding similar to that reported by Eraslan (57). The analysis of the causes suggests that, cancer information is highly complex and uncertain, with numerous technical terms and intricate medical knowledge spanning disease diagnosis, treatment selection, and prognosis evaluation. This complexity challenges patients’ comprehension and processing abilities, fostering confusion and fear that intensify negative emotions. Meanwhile, the diversity and inaccuracy of modern information channels further compound information overload. In the digital era, patients can access information through various channels such as the internet, social media, and patient - support groups. However, these platforms contain a wealth of unverified information, with some content potentially being false or exaggerated. Patients often lack the ability to discern the accuracy of this information and are easily misled, leading to unnecessary anxiety and panic. This phenomenon aligns with the Limited Capacity Model of Motivated Mediated Message Processing (15, 16), This model emphasizes that individuals’ information processing systems have limited cognitive capacity, and that information overload occurs when the amount of information exceeds an individual’s information processing threshold, leading to impaired information processing efficiency and subsequently having a negative impact on their physical and mental health, thereby confirming the core mechanism of this theory. In addition, information overload can also lead to decision-making conflicts, making it difficult for patients to determine which treatment plan is most suitable for their condition. This uncertainty in decision-making can significantly increase psychological stress, thereby exacerbating negative emotions (31).

The mediation effect test shows that FoP partially mediates between CIO and negative emotions. Specifically, CIO creates a massive and complex information environment, increasing the difficulty for patients to screen for effective information related to their condition. This information processing obstacle directly reinforces patients’ FoP, which in turn amplifies their concerns about the deterioration of their condition, ultimately inducing negative emotions. This mediating mechanism can be further explained by cognitive appraisal theory (58), this theory suggests that stress arises from an individual’s cognitive evaluation of an event, involving two stages: primary appraisal and secondary appraisal. During primary appraisal, the individual assesses whether the event poses a potential threat or harm. When lung cancer patients meet with CIOs, they find it difficult to quickly understand the disease information due to the large amount of technical terms and complex treatment details involved. This can lead to uncertainty about their condition, which they may perceive as a potential threat, thereby triggering FoP (59). The secondary assessment stage is an individual assessment of the ability to cope with threats. Due to a lack of medical knowledge and information discernment skills, as well as a lack of professional guidance, patients feel helpless in the face of negative information about the risk of disease progression, which amplifies FoP and triggers negative emotions (60).

The moderated mediation analysis revealed that, after controlling for employment status, surgery, radiotherapy, cancer stage, and number of chronic diseases, the interaction between CIO and social support on negative emotion in newly diagnosed lung cancer patients was non - significant. This indicates that social support does not significantly mediate the relationship between CIO and negative emotions. This may stem from CIO being influenced by multiple factors such as information source, content, and individual cognitive capacity, with its core issue being cognitive overload requiring informational support (61), rendering conventional social support less effective. Research indicates that at initial diagnosis, patients’ strong desire for disease information and high anxiety may reduce their perception of social support (62). Furthermore, recent studies show that personality traits are related to psychological states. Individuals with high neuroticism are more likely to experience negative emotions (63), and the interaction between cancer diagnosis and personality traits can affect patients’ psychological responses to the disease (64). These factors can interfere with the moderated mediation model. Future research may further explore the impact of the interaction between CIO and social support on negative emotions by increasing sample size, altering sample characteristics, or refining the categorization of social support types.

Concurrently, FoP and Social Support exhibit a significant interaction effect on Negative Emotion among newly diagnosed lung cancer patients, social support mitigated the effect of FoP on negative emotions and weakened the mediating effect of FoP between CIO and negative emotions. In low social support, FoP had the greatest effect on negative emotions, and this effect gradually decreased as social support levels increased. Furthermore, the mediating effect of FoP also decreased as social support increased. This conclusion is consistent with the Buffering Effect Model of social support, whereby social support mitigates the negative impact of stress on physical and mental health by regulating the cognitive evaluation and coping process of stressful events (26). When patients have low levels of social support, they lack sufficient social resources to cope with the psychological stress caused by CIO, making them more susceptible to FoP. Conversely, high levels of social support provide more emotional and informational support, helping patients effectively alleviate their fears about disease progression (65), Enhancing its coping effectiveness, thereby significantly reducing the association between FoP and negative emotions and its mediating effect between CIO and negative emotions.

Based on the findings, we propose the following recommendations. First, develop a culturally adapted negative emotion screening protocol for Chinese lung cancer patients and integrate it into routine clinical monitoring. Focus on critical stages including initial diagnosis, treatment plan adjustments, postoperative recovery, and advanced palliative care to enable timely psychological intervention. Implement stratified psychological intervention strategies for patients with advanced disease, multiple comorbidities, or undergoing radiotherapy to address diverse psychological support needs. Second, optimize information delivery by adopting a phased, stepwise approach tailored to patients’ cognitive and psychological capacity. At the time of initial diagnosis, prioritize conveying core information such as key diagnostic results and treatment objectives. As treatment progresses, progressively supplement details regarding treatment risks and prognosis in line with patients’ actual needs and therapeutic advancements. Healthcare institutions should actively develop and promote reliable patient education platforms that consolidate authoritative cancer information resources, thereby minimizing patients’ exposure to misinformation. Finally, to enhance social support for cancer patients, healthcare professionals are recommended to assess patients’ existing social support networks and actively encourage their expansion through participation in mutual support groups and community activities. Additionally, the government should establish dedicated cancer support services, including 24/7 helplines staffed by professional operators and counselors, to provide timely psychological assistance, information consultation, and resource linkage for patients and their families. Furthermore, special charitable funds should be developed to offer employment assistance and financial support to unemployed or economically disadvantaged patients, thereby alleviating their financial burdens.

## 5 Conclusion

This study focuses on a population of newly diagnosed lung cancer patients, systematically exploring the relationships between cancer information overload (CIO), fear of disease progression (FoP), social support, and negative emotions. It reveals a direct association between CIO and negative emotions, clarifies the mediating role between CIO and negative emotions, and identifies the moderating effect of social support, which significantly attenuates the positive influence of FoP on negative emotions. The study’s conclusions emphasize the importance of information management and social support, providing a theoretical basis for effectively establishing precise psychological care models to improve patients’ mental health outcomes in the early stages of the disease, and offering new directions for future research and practice in related fields.

## 6 Limitations

This study has the following limitations, which need to be improved in future work. First, the cross-sectional design limits the inference of causal relationships between variables. In the future, longitudinal tracking or randomized controlled trials should be used to further verify the temporal path through which CIO influences negative emotions via FoP, as well as other potential moderating factors such as digital health literacy. Secondly, despite controlling for confounding variables such as surgery, radiotherapy, and cancer staging, variables such as patients’ nutritional status, severity of sleep disorders, financial toxicity, stigma, coping strategies, health literacy, and patients’ subjective perception of disease uncertainty may all be significantly associated with anxiety and depression but were not included in this study. Future studies should incorporate potential influencing factors spanning clinical, psychological, and social dimensions to enhance the explanatory validity of mechanisms underlying negative emotions and provide evidence for precise interventions.

Third, this study’s sample was drawn from three hospitals in a Chinese city and comprised solely lung cancer patients, it remains uncertain whether the findings can be extrapolated to other cancer patients. Furthermore, cultural, healthcare system, and stigma-related factors may also limit its external validity. Concurrently, the sample is characterized by older age, a male majority, and predominantly rural origins, which may compromise representativeness. Future studies should expand the sampling scope to include multi-center, community, and primary healthcare institutions to enhance the representativeness of the samples.

Finally, negative emotions were assessed solely using the HADS to measure anxiety and depression, employing a rather limited measurement tool that failed to encompass broader mental health indicators such as loneliness, perceived stress, subjective well-being, and life satisfaction. Future studies should integrate multidimensional psychological scales and biomarkers to establish a more comprehensive mental health assessment framework, thereby comprehensively evaluating patients’ negative emotions.

## Data Availability

The raw data supporting the conclusions of this article will be made available by the authors, without undue reservation.
